# Long noncoding RNA *LINC00518* contributes to proliferation and metastasis in lung adenocarcinoma via the *miR-335-3p/CTHRC1* Axis

**DOI:** 10.1038/s41420-022-00905-w

**Published:** 2022-03-04

**Authors:** Ruoyi Shen, Xin Cai, Dan Shen, Ruochen Zhang, Weijie Zhang, Yang Zhang, Yue Li, Anqi Wang, Yuanyuan Zeng, Jianjie Zhu, Zeyi Liu, Jian-an Huang

**Affiliations:** 1 Department of Pulmonary and Critical Care Medicine, the First Affiliated Hospital of Soochow University, Suzhou, 215006 China; 2 Institute of Respiratory Diseases, Soochow University, Suzhou, 215006 China; 3 Suzhou Key Laboratory for Respiratory Diseases, Suzhou, 215006 China

**Keywords:** Non-small-cell lung cancer, Prognostic markers

## Abstract

Long intergenic nonprotein coding RNA 518 (LINC00518) is recognized to impart cancer proliferation and metastasis in lung adenocarcinoma (LUAD). However, the study about the relationship between LINC00518 and LUAD is shallow so far. In our work, LINC00518 was predicted to be a negative regulator in LUAD based on the TCGA database. It was further verified that the cell proliferation, colony formation, migration, and invasion of LUAD could be obviously inhibited by the knockdown of LINC00518. Moreover, miR-335-3p/CTHRC1 axis was intensively possible to be a critical regulator in the effect of LINC00518 on LUAD via visual ceRNA network. Importantly the progress of LUAD was relevant to the active CTHRC1 which was realized by the target of LINC00518 to miR-335-3p. Furthermore, the knockdown of LINC00518 exhibited a synergistic effect with VS6063, an inhibitor of FAK protein, in the suppression of LUAD indicating that miR-335-3p/CTHRC1 axis was potentially exploitable as a targeted intervention to integrin β3/FAK signal pathway in LUAD. All the collective results demonstrated that LINC00518 could be a promising biomarker of the prognosis of LUAD and possibly a therapeutic target via miR-335-3p/CTHRC1 axis.

## Introduction

In nowadays society, lung cancer remains at the top of the list of tumor-related deaths around the world, and its incidence has increased steadily by 10% annually [1, 2]. Lung cancer could be divided into two histological subtypes: nonsmall cell lung cancer (NSCLC) and small cell lung cancer (SCLC). NSCLC accounts for around 85% of lung cancers, and includes adenocarcinoma, squamous cell carcinoma, adenosquamous carcinoma, and large cell carcinoma; SCLC accounts for 15% of lung cancers [3]. Lung adenocarcinoma (LUAD) is one of the major types of lung cancer [4]. Therefore, the mechanism of proliferation, metastasis, and drug resistance of LUAD should be explored extremely urgently.

Long noncoding RNAs (lncRNAs) are noncoding RNAs of more than 200 nucleotides, that can be used as biomarkers for the diagnosis and prognosis of numerous human tumors [5]. Long intergenic nonprotein coding RNA 518 (LINC00518), has been known to promote and up-regulate cancer cell proliferation and metastasis in cervical cancer, breast cancer, and malignant melanoma [6, 7]. It also makes a contribution to chemotherapeutic drug resistance in prostate cancer and breast cancer [8, 9]. In addition, it could induce radioresistance by regulating glycolysis in melanoma [10]. Some researchers have shown that LINC00518 acts as an oncogene to facilitate tumor progression in NSCLC by regulating an RNA-based network [11]. Nevertheless, the accurate role and molecular mechanism of LINC00518 in LUAD are still undetermined.

Certain specific lncRNAs, substantial studies have demonstrated that, may serve on competitive endogenous RNAs (ceRNAs) in tumorigenesis and development [12]. Some researches have been made to demonstrate that, in melanoma, prostate cancer, and breast cancer, LINC00518 plays a similar role [8, 9, 13]. Thus, we conjectured that LINC00518 might be the same in LUAD and constructed the ceRNA network of LINC00518.

Collagen triple helix repeat containing 1 (CTHRC1), what we know about, is a chondrocyte-specific, secreted glycoprotein that was initially discovered in a rat model of balloon-injured arteries [14]. It is frequently detected in several solid tumors that the expression of CTHRC1 is high, such as breast ductal carcinoma, hepatocellular carcinoma, gastric cancer, colorectal cancer, and melanoma [15, 16]. It has been reported that high expression of CTHRC1 was meaningfully correlated with metastasis in patients with NSCLC and that over-expression of CTHRC1 might be involved in tumor poor prognosis and angiogenesis in LUAD [17].

As a sort of anchoring junction, focal adhesion dominantly is mediated by integrins that integrate the surrounding extracellular matrix (ECM) with the actin cytoskeleton [18]. As the kernel constituents of focal adhesion, 24 transmembrane αβ heterodimers constituted the integrin family, which generated from selective noncovalent unions between 18 α and 8 β subunits. Integrin β3, among them, plays a predominant role and is significantly associated with malignant phenotypes of tumors [19].

This study aims to identify that LINC00518 sponges miR-335-3p to activate CTHRC1 transcription and integrinβ3/FAK signaling, which are required for LUAD proliferation and metastasis. We also showed that LINC00518 is highly expressed in LUAD tissue and plays a critical role in prognosis, which could serve as a predictive biomarker and potential therapeutic target for LUAD.

## Results

### LINC00518 is upregulated in lung adenocarcinoma (LUAD) tissues and plays a significant role as a prognostic factor

**Fig. 1:**
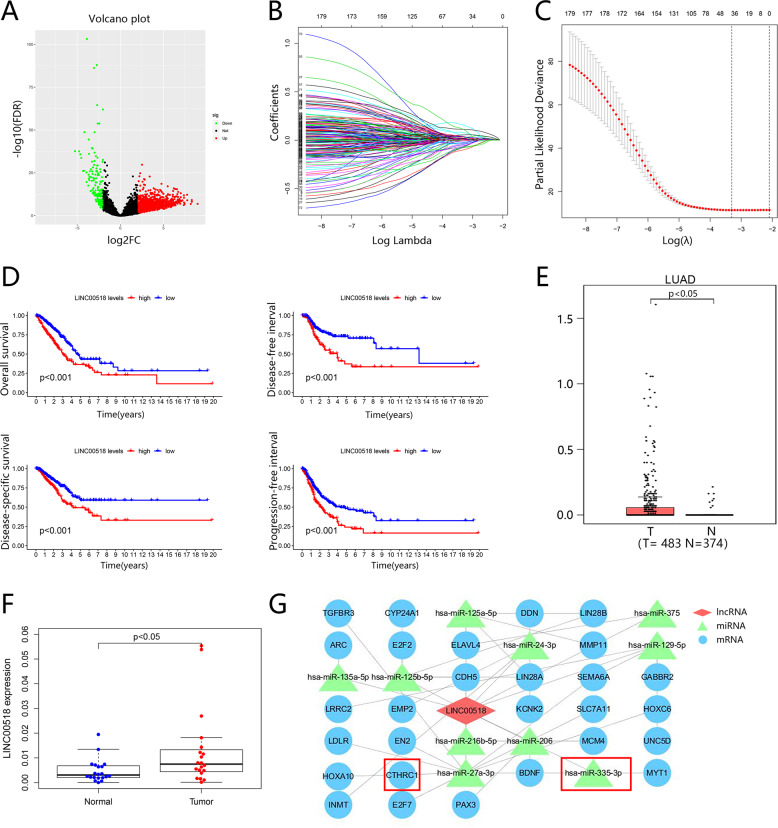
Up-expressed LINC00518 was a venturesome factor for the survival of LUAD. **A** Differential expression of lncRNAs in LUAD was exhibited by volcano map. **B** Lambda of the LASSO regression of differentially expressed lncRNAs. **C** The minimum lambda included 39 lncRNAs. **D** The OS, PFS, DFS, and DSS of LUAD patients with high or low expression of LINC00518. Computed p-value by using the log-rank test. **E** LINC00518 is upregulated in LUAD compared with normal tissues. **F** The expression of LINC00518 was tested by qPCR in 20 paired LUAD tissues and their corresponding adjacent noncancerous lung tissues. **G** Cytoscape provided the visual interaction of LINC00518 − miRNA−target gene.

To screen potential oncogenic lncRNAs in LUAD, we compared lncRNA expression in LUAD and normal tissues, which were downloaded from the TCGA database. And the volcano map showed all differentially expressed lncRNAs in LUAD from TCGA (Fig. 1A). Then we used COX and LASSO regression to analyze all differentially expressed lncRNAs. The lambda model of the LASSO regression is shown in Fig. 1B. We chose the minimum lambda, and 39 lncRNAs were selected (Fig. 1C and Supplementary Table S3). Next, we detected the expression of 39 lncRNAs and found that LINC00518 expressed higher in lung adenocarcinoma tissues by using the GEPIA (http://gepia.cancer-pku.cn/) (Fig. 1E). To verify this conclusion, we analyzed the LINC00518 levels in 20 LUAD tissues and para-carcinoma tissue samples and found the same results (Fig. 1F). We analyzed the overall survival (OS), disease-free survival (DFS), disease-specific survival (DSS), and progression-free survival (PFS) of 483 LUAD patients with high or low LINC00518 expression. The collective results concerning the progression of LUAD exhibited that the poorer prognosis was closely accompanied with the higher expression of LINC00518. (Fig. 1D)

### The ceRNA for LINC00518

It is commonly reported that the proliferation of some other human tumors is aggravated through miRNA or mRNA controlled by upstream LINC00518. For the exploration of which miRNA or mRNA functions likewise in lung adenocarcinoma, the software Cytoscape (v3.6.0) was used to display a visual network about the interrelationship of ceRNA for LINC00518 (Fig. 1G). Surprisingly, miR-335-3p was showed a strong relevance to LINC00518, whose corresponding miRNA, miR-335-5p, was found downregulated in LUAD in our previous research [20]. Moreover the target gene of miR-335-3p was predicted to be CTHRC1 which was increased in LUAD in many studies [21]. Verified by this work as follows the predicted network of ceRNA showed an excellently possible miR-335-3p / CTHRC1 axis in LUAD regulated by LINC00518.

### LINC00518 knockdown suppresses LUAD cell proliferation, colony formation, migration, and invasion, and arrests the cell cycle

**Fig. 2:**
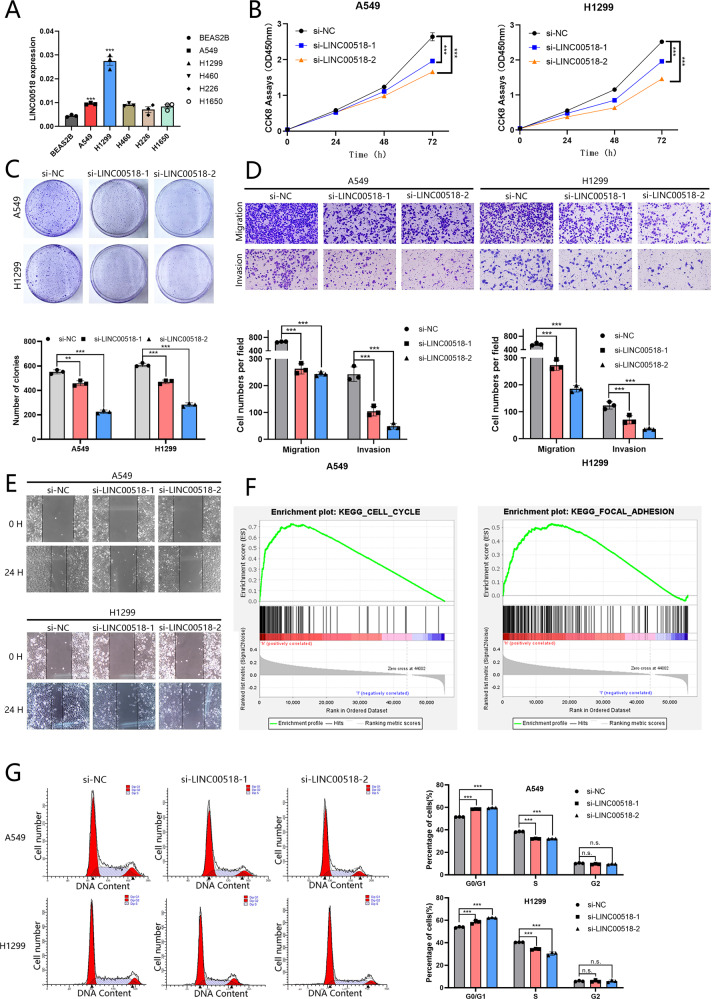
Knockdown of LINC00518 suppressed cell proliferation and metastasis, and reduced viability. **A** Expression level of LINC00518 in human bronchial epithelial cells (BEAS-2B) and lung cancer cell lines(A549, H1299, H460, H226, H1650) detected by qRT–PCR. **B**, **C** Cell proliferation was detected by CCK-8 assay and Colony formation analysis. **D**, **E** The cell metastasis capability was tested by Transwell assay and Wound-healing assay. **F** Kegg pathway analysis identified that LINC00518 influenced the cell cycle and focal adhesion. **G** Cell cycle distributions were analyzed in A549/H1299 cells. All results indicate SD. (*P < 0.05, **P < 0.01, ***P < 0.001).

Lung adenocarcinoma cells (A549 and H1299) expressed higher LINC00518 levels than human bronchial epithelial cells (BEAS-2B) (Fig. 2A). After establishing siRNA efficacy, the Cell Counting Kit-8 assay and colony formation assays identified that the proliferation was expressively decreased in LINC00518-reduced cells compared to that in control (Fig. 2B, C). Next, we had access to the wound-healing and transwell assays to measure the impression of LINC00518 on the migration and invasion of LUAD cells, respectively (Fig. 2D, E). To determine how LINC00518 knockdown suppressed cell proliferation in LUAD cells, we conducted KEGG pathway analysis using the TCGA database by Gene Set Enrichment Analysis (GSEA) 4.1.0 software. The results showed that LINC00518 may be essential to the cell cycle and focal adhesion (Fig. 2F). Then, we detected that the proportion of cells in the S phase was efficiently lower and in the G0/G1 phase was efficiently higher in LUAD cells by the flow cytometry (Fig. 2G).

### MiR-335-3p is directly targeted by LINC00518 and suppresses the LUAD cell proliferation, colony formation, migration, and invasion

**Fig. 3:**
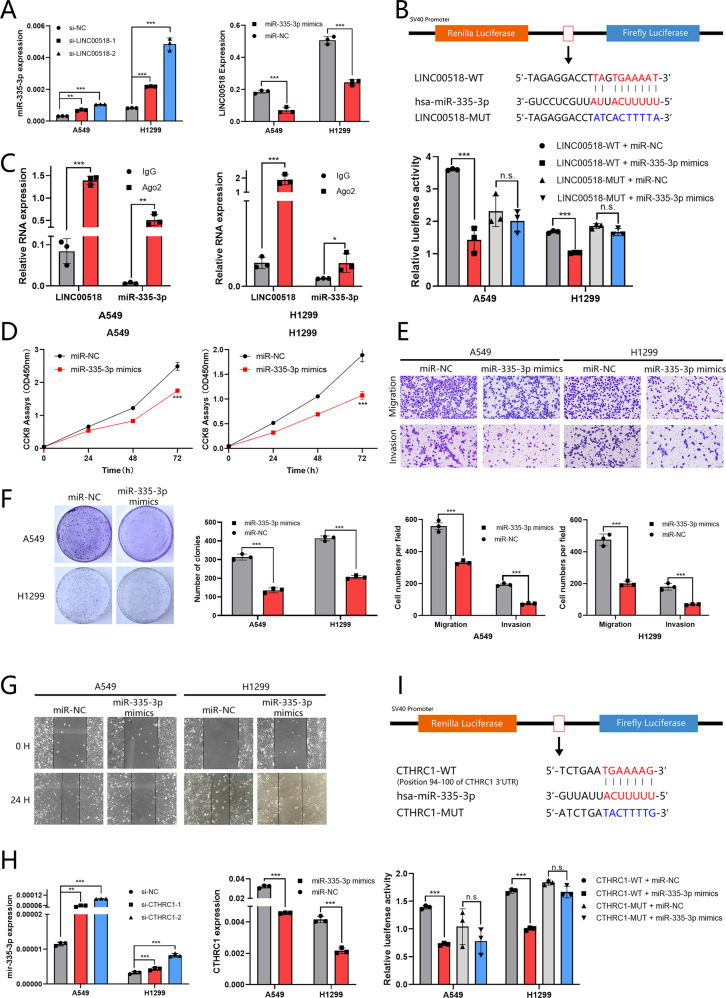
LINC00518 directly targets miR-335-3p, promoting CTHRC1 expression and miR-335-3p suppresses LUAD cell proliferation and metastasis. **A**, **H** Detection of the expression of miR-335-3p in A549/H1299 cells transfected with LINC00518 siRNA, CTHRC1 siRNA, or NC and the expression of LINC00518 or CTHRC1 of cells transfectied with miR-335-3p mimics or NC. **B**, **I** The dual-luciferase assay showed that the relative dual-luciferase activity of the LINC00518-WT or CTHRC1-WT group was directly inhibited by miR-335-3p mimics. **C** The direct interaction between LINC00518 and miR-335-3p was verified by RIP assay. **D**, **F** Cell proliferation and colony formation of A549/H1299 cells was inhibited by miR-335-3p mimics. **E**, **G** Transwell assay and Wound-healing assay showing that miR-335-3p mimics significantly inhibited the ability of cell metastasis in A549/H1299 cells. All results indicate SD. (*P < 0.05, **P < 0.01, ***P < 0.001).

First, in A549 and H1299 cells, we detected the expression of LINC00518 transfected with miR-335-3p. The level of LINC00518, consistent with the expression of miR-335-3p, was downregulated in cells transfected with the miR-335-3p mimics, as detected by using qRT–PCR (Fig. 3A). To make sure of this prediction, we next constructed LINC00518 luciferase plasmids containing the wild-type and mutant miR-335-3p binding sites in Fig. 3B. Moreover, the RIP assay further verified the direct interaction between LINC00518 and miR-335-3p (Fig. 3C).

We induced overexpression of miR-335-3p in LUAD cells by using mimics, and studied their impressions on cell growth by CCK-8 assays and colony formation assays (Fig. 3D, F). To determine the effects of miR-335-3p transfection in A549/H1299 cells, the wound-healing assay and transwell assays was performed in Fig. 3E, G.

### CTHRC1 is regulated by miR-335-3p and increases the proliferation, migratory and invasive abilities of LUAD cells

The level of CTHRC1, corresponding with miR-335-3p, was downregulated in cells transfected with the miR-335-3p mimics, as determined via qRT–PCR (Fig. 3H). To determine this prediction, we constructed the CTHRC1 wild-type 3’-UTR and CTHRC1 MUT 3’-UTR and performed the dual-luciferase reporter assay in A549 and H1299 cells (Fig. 3I).

**Fig. 4:**
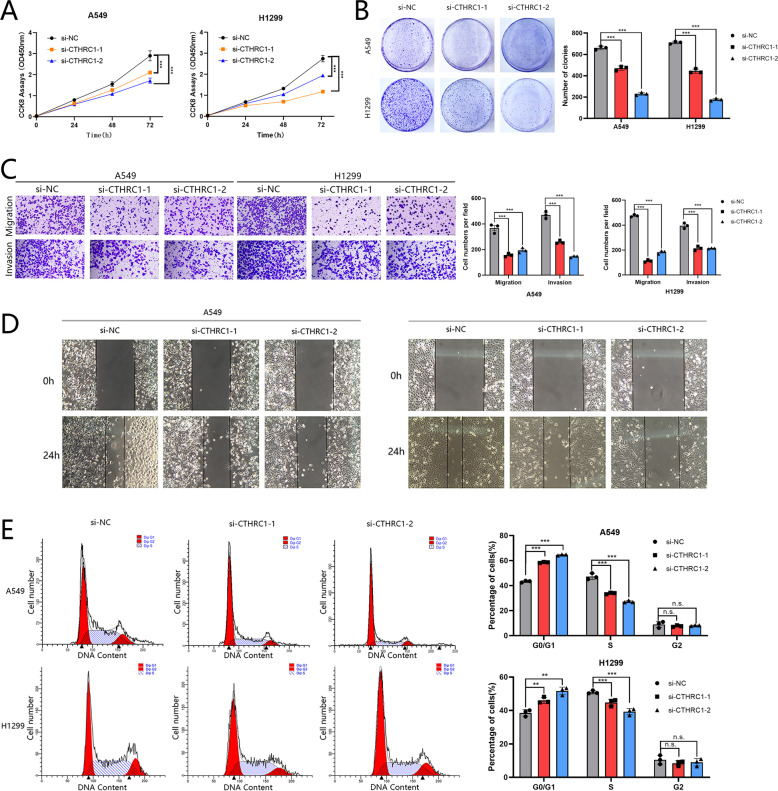
Knockdown of CTHRC1 suppressed cell proliferation and metastasis, and reduced viability. **A**, **B** The capability of cell proliferation of A549/H1299 cells transfected with si-CTHRC1 or NC was detected by CCK-8 assay and colony formation analysis. **C**, **D** Transwell assay and Wound-healing assay showing that knockdown of CTHRC1 in A549/H1299 cells inhibited cell metastasis ability. **E** Cell cycle distributions were analyzed in A549/H1299 cells. All results indicate SD. (*P < 0.05, **P < 0.01, ***P < 0.001).

We established the CTHRC1-targeting small interfering RNA (siRNA) in A549 and H1299 cells. The growth of the control cells, determined by CCK-8 and colony formation assays, was observably promoted compared with that of the cells with low CTHRC1 expression (Fig. 4A, B). Moreover, knockdown of CTHRC1 suppressed the migratory and invasive abilities of A549 and H1299 cells were shown by wound healing and transwell assays (Fig. 4C, D). The flow cytometry results also showed that CTHRC1 can promote proliferation by affecting the cell cycle in LUAD cells (Fig. 4E).

### CTHRC1 could affect integrin β3/FAK signaling and LINC00518 might be a potential synergistic therapeutic target of the FAK-inhibitor VS-6063

**Fig. 5:**
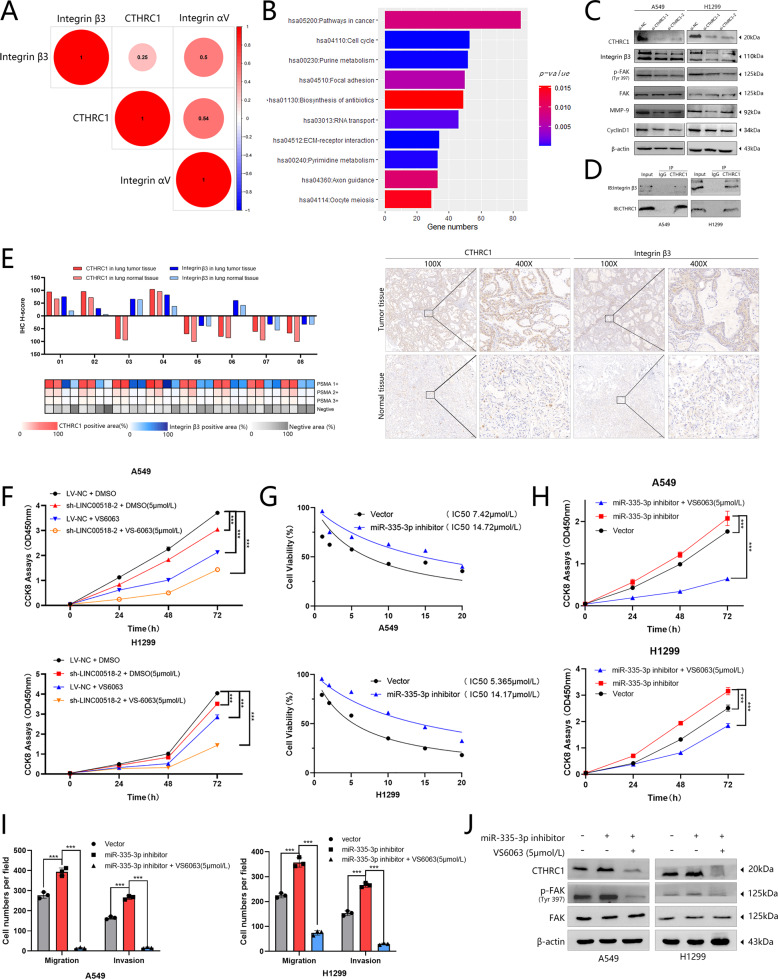
CTHRC1 could affect integrinβ3/FAK signaling. **A** The correlation of CTHRC1 and integrin αVβ3 from the TCGA database. **B** Pathway analysis and functional analysis was separately according to TCGA database and DAVID database. **C** Western blotting showed that integrin β3, p-FAK, FAK, MMP-9, and CyclinD1 protein levels were regulated by CTHRC1. **D** Co-immunoprecipitation of CTHRC1 and integrin β3 in LUAD cells exposed that endogenous integrin β3 in LUAD cells was immunoprecipitated by CTHRC1 antibody. **E** The co-expression between CTHRCI and integrin β3 was shown by IHC of sections from the patients (scale bar, 100 μm, 20 μm). **F** CCK-8 assays were applied to detect the cell with VS6063 (5 μmol/L) had notably lower capability of cell proliferation than the control cells with DMSO (5 μmol/L). **G** The IC50 of A549 and H1299 cells, which inhibited miR-335-3p. **H** The IC50 of A549 and H1299 cells, which inhibited miR-335-3p. **I** CCK-8 assay showed the proliferation of A549/H1299 cells that inhibited miR-335-3p transfection with VS6063 (5 μmol/L). **I** Transwell assays showed that miR-335-3p inhibited cells migration and invasion following transfection. **J** Western blotting analysis showed that CTHRC1 and p-FAK protein levels were induced by a FAK-inhibitor when miR-335-3p was inhibited. All results indicates SD. (*P < 0.05, **P < 0.01, ***P < 0.001).

First, the expression data of mRNA in LUAD was downloaded from the TCGA database, and we implemented an analysis of co-expression between CTHRC1 and integrin (Fig. 5A). Then, put the information into the DAVID database (https://david.ncifcrf.gov/) for functional analysis, including the data from TCGA database, the analysis of co-expression performed before with CTHRC1 coefficient of 0.3, *P* < 0.05, and the selected genes. It could be seen that CTHRC1 could be clustered in cell cycle and focal adhesion pathways (Fig. 5B). To determine whether CTHRC1 could affect the integrin β3/FAK signaling or not, we used Western blotting analysis (Fig. 5C). It could be revealed by co-immunoprecipitation of integrin β3 and CTHRC1 that endogenous integrin β3 of LUAD cells was immunoprecipitated by CTHRC1 antibody (Fig. 5D). Then we used the H-Score to judge the expression of CTHRC1 and integrin β3. The results of IHC staining showed a correlation of expression between them in tissues (Fig. 5E, Supplementary Table S5 and Supplementary Fig. S2).

To further explore the clinical significance of the study, we examined the IC50 of VS-6063, the FAK inhibitor, in A549 and H1299 cells, and the results indicated that the miR-335-3p inhibitor could elevate the IC50 of VS-6063 (Fig. 5G). CCK-8 assays also showed that the cells with VS-6063(5 μmol/L) had significantly lower proliferation ability (Fig. 5F, H). In addition, we used Transwell assays to show that VS-6063 could suppress the migratory and invasive abilities of LUAD cells when miR-335-3p was inhibited (Fig. 5I and Supplementary Fig. S1A). FAK signaling, which is the drug target of VS-6063, was induced by Western blotting analysis (Fig. 5J).

### Effect of LINC00518 in LUAD cells in vivo

**Fig. 6:**
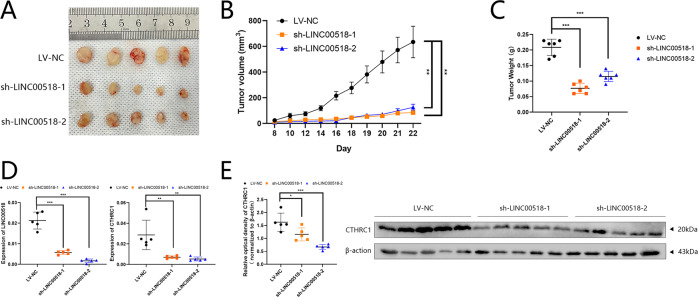
Knockdown of LINC00518 inhibits LUAD tumor growth in vivo. **A**–**C** The transplantation tumor experiments showed that repression of LINC00518 expression by shLINC00518 plasmid reduced the tumorigenic ability and tumor volume of A549 cells. **D** The expression level of LINC00518 and CTHRC1 in tumor tissues of mice was detected by qRT–PCR. **E** As is shown in Western blotting that LINC00518 expression or treatment of the cells with LV-NC decreased CTHRC1 protein expression in tumor tissues of mice. All results indicate SD. (*P < 0.05, **P < 0.01, ***P < 0.001).

First, a xenograft mouse model was used to confirm the effect of LINC00518 in LUAD cells in vivo. As shown in illustration (Fig. 6A, B), tumors formed by LINC00518 knockdown cells were significantly smaller in size than those formed by the contrast cells. Tumor weights, in accordance with these results, were found to be lighter in cells with LINC00518 knockdown (Fig. 6C). Next, we resected the tissues from the xenograft tumors, and analyzed them to verify LINC00518, and CTHRC1 by Western blotting and qRT–PCR (Fig. 6D, E).

### Down-regulation of miR-335-3p reverses the inhibition of cell proliferation, migration, invasion, and colony formation induced by LINC00518 knockdown

**Fig. 7:**
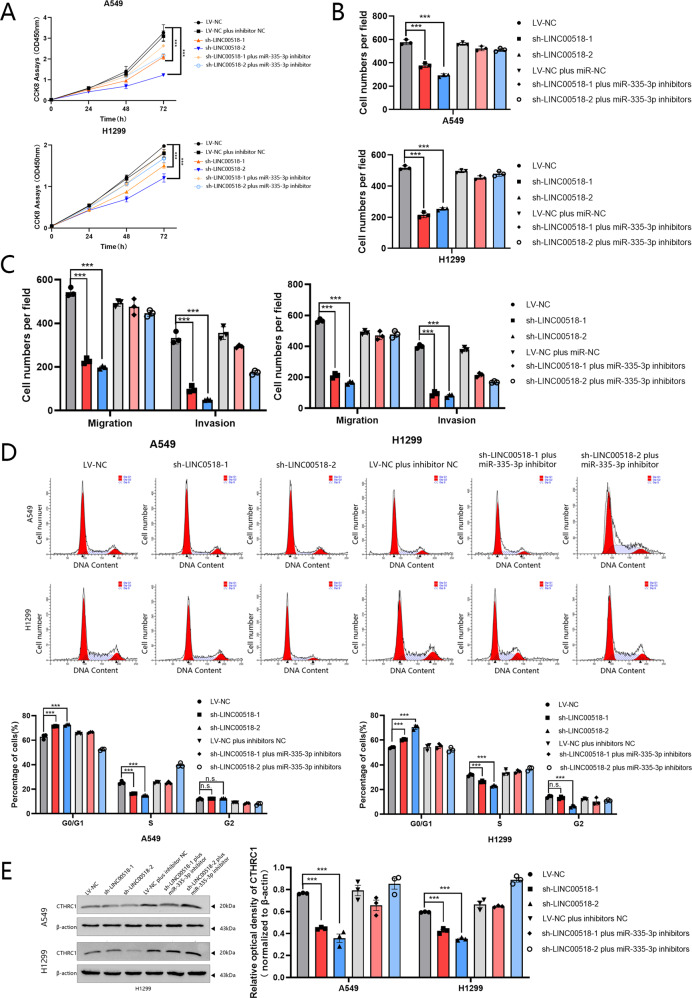
LINC00518 promotes LUAD cell proliferation and metastasis through the miR-335-5p/CTHRC1 axis. **A**–**B** CCK-8 assay and colony formation analysis were applied to detect the proliferation of A549/H1299 cells transfected with LINC00518 shRNA, LINC00518 shRNA plus miR-335-3p inhibitor, or the control. **C** The effect on LUAD cell migration and invasion following transfection is tested by transwell assay. **D** Cell cycle distributions were analyzed in A549 and H1299 cells by flow cytometry. **E** Western blots identified the protein expression changes in LINC00518 shRNA and LINC00518 shRNA plus miR-335-3p inhibitor transfected LUAD cells. β-actin was used as a control. All results indicate SD. (*P < 0.05, **P < 0.01, ***P < 0.001).

**Fig. 8:**
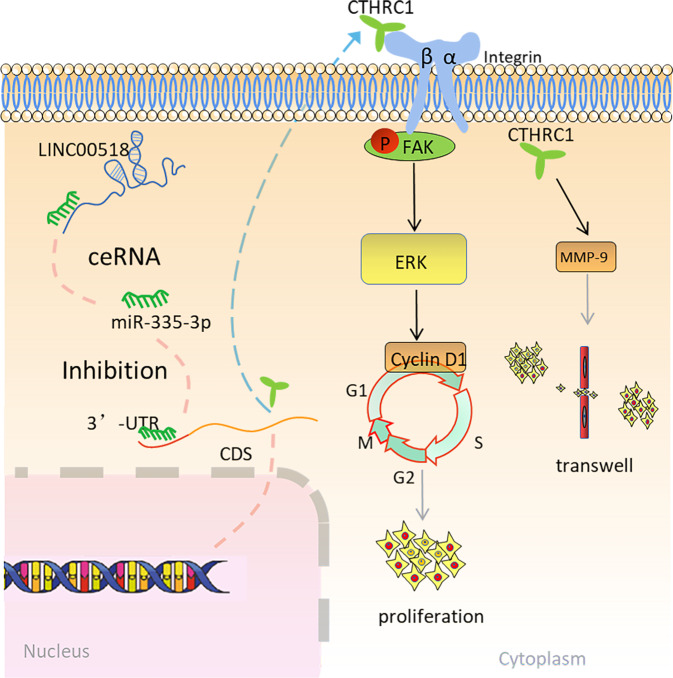
The model of this study. The model of the LINC00518/miR-335-3p/CTHRC1 axis is in the control of the integrin β3/FAK signaling pathway.

Transfection of A549 and H1299 cells with sh-LINC00518-reduced cell proliferation while inhibiting miR-335-3p reversed this effect (Fig. 7A). Additionally, it was identified that the cell survival capability was remarkably decreased in the LINC00518-silenced cells, by colony formation assays, while this negative impression on cell survival could be reversed by the low expression of miR-335-3p (Fig. 7B and Supplementary Fig. S1B). Besides, LINC00518 got in touch with regulating invasion and migration in LUAD cells, suggested by Transwell assays, and miR-335-3p inhibitors reversed LINC00518 knockdown-induced suppression of migration and invasion (Fig. 7C and Supplementary Fig. S1C). The flow cytometry results also showed that the impact on the cell cycle of LINC00518 knockdown in A549 and H1299 cells could be reversed by miR-335-3p inhibitors (Fig. 7D). Knockdown the expression of LINC00518 in A549 and H1299 cells decreased protein levels of CTHRC1 were confirmed by Western blotting, while miR-335-3p inhibitors reversed this effect (Fig. 7E). A schematic of this search is shown in Fig. 8.

## Discussion

Located on human chromosome 6p24.3, the expression of long intergenic nonprotein coding RNA 518 (LINC00518) is significantly upregulated in some tumor tissues [6, 8, 13]. LINC00518, indicated from the published studies, might be a crucial cancer gene and take an indispensable part in the occurrence and development of tumors through various biological mechanisms. Nevertheless, the function of LINC00518 in the progression of lung adenocarcinoma has not yet been clarified.

As crucial regulators in multiple cellular processes associated with tumorigenesis and metastasis, in the past decades, lncRNAs have drawn great much attention and interest [22, 23]. In the present study, we noted that LINC00518 expression was meaningfully upregulated in LUAD tissues contrasted with normal lung tissues and that patients had a markedly poor prognosis in terms of OS, PFS, DFS, and DSS with high expression of LINC00518. This shows that LINC00518 might be a potential biomarker for the diagnosis and prognosis and a molecular target of patients with LUAD. Moreover, the proliferation and clonogenicity of LUAD cells could be increased by LINC00518, migration and invasion could be induced, and the cell cycle could be affected. It might suggest that LINC00518 serves as an oncogene in LUAD cells.

Serving as miRNA sponges, LINC00518 regulates the target genes of miRNAs, basically localized in the cytoplasm, thereby inhibiting miRNAs′ functions [8–10]. Hence, we investigated the miRNAs that bind to LINC00518. It was verified in this study that LINC00518 could directly target miR-335-3p, which, in turn, target CTHRC1. In other words, LINC00518 upregulates the expression of CTHRC1 by binding competitively to miR-335-3p.

Next, we demonstrated that LINC00518 could specifically inhibit the expression of miR-335-3p or not. Our previous study showed that upregulating miR-335-5p expression could inhibit the proliferation and migration of NSCLC cells [20]. Accordingly, we considered that miR-335-3p, homologous with miR-335-5p, played the same role in LUAD. To verify the specific function of miR-335-3p in LUAD cells, we performed malignant phenotype assays. We detected that miR-335-3p could take a part in an antioncogene by inhibiting the activity of CTHRC1 in LUAD. Our investigation results showed that miR-335-3p might serve as a tumor suppressor, which has tremendous significance in the development of new targeted therapies for LUAD.

Although the function of CTHRC1 in LUAD is well-known, the mechanisms remained unclear. Research conducted before showed that expression of CTHRC1 is associated with prognosis and can act as an important predictor of progression-free survival (PFS) and overall survival (OS) in LUAD [21]. CTHRC1 could induce the invasion ability of NSCLC by upregulating MMP-7/MMP-9 [24]. In our study, we verified that in LUAD cells the ability of cell proliferation, migration, and invasion could be increased by CTHRC1.

Integrins could be classified into four, including collagen receptors, laminin receptors, leukocyte-specific integrins, and receptors recognizing Arg-Gly-Asp (RGD) peptide motifs [25, 26]. There are reports of CTHRC1, which could accelerate migration and adhesion by upregulating the expression of the integrin β family, and the phosphorylation of focal adhesion kinase (FAK) in ovarian cancer, hepatic carcinoma, and pancreatic cancer [27–29]; however, it has not been found in lung cancer yet. As a crucial cell movement regulator, FAK could be stimulated via phosphorylation by transmembrane integrins and varieties of growth factors, connecting to the formation and turnover of focal adhesions, which then phosphorylated and induce downstream pathway signaling [30, 31]. It has been reported that ligation of αVβ3 and αvβ5 integrins could mediate FAK activity [32]. In our previous research, integrin αVβ3 could promote cell proliferation in NSCLC by activating the downstream FAK/AKT and ERK signaling pathways [33]. We used online database functional analysis to find that LINC00518 and CTHRC1 could be clustered on FAK signaling and CTHRC1 co-expressed with integrin β3 in LUAD.

Conformably, our research indicated that the low-expression of CTHRC1 influences the expression of integrin β3, and the phosphorylation of FAK. Moreover, we gave a piece of evidence that CTHRC1 physically interacts with integrin β3, which further attests to the mechanism of CTHRC1 in LUAD. In the study, the results showed that CTHRC1 could regulate FAK signaling by interacting with integrin αVβ3 complexes. Whereas, it remains to further detect the complicated mechanism of the CTHRC1-integrin complex.

Previous studies have demonstrated that targeting the integrin β3/FAK signaling could enhance the anti-tumor activity and attenuate cancer metastasis in melanoma, endometrial cancer, NSCLC, and ESCC [34–36]. Consequently, the FAK-inhibitor VS-6063 was used to verify whether the inhibition of FAK phosphorylation would work in conjunction with the knockdown of LINC00518 on LUAD cell proliferation. Moreover, overexpression of CTHRC1 could reverse the LUAD cell growth inhibition induced by VS6063 treatment. Hence, we proved that CTHRC1 can affect integrin β3/FAK signaling and broaden the application of the FAK-inhibitor VS-6063 in LUAD which might shed light on clinical treatment. Interestingly, the FAK-inhibitor reduced the expression of CTHRC1 when miR-335-3p was inhibited and CTHRC1 was over-expressed. We suspect that there is positive feedback between CTHRC1 and FAK signaling. In summary, our results provide the first evidence that CTHRC1 interacts with integrin β3 and accelerates FAK phosphorylation in LUAD.

To sum up, we clarified the clinical significance and biological functions of a cytoplasmic lncRNA, LINC00518, in LUAD. LINC00518, as a molecular sponge to regulate CTHRC1, promoted LUAD growth and migration through integrin-mediated focal adhesion signaling. Furthermore, the expression of LINC00518 is high in LUAD samples than in normal, and the expression of LINC00518 is an indicator of prognosis in LUAD patients. The LINC00518 /miR-335-3p /CTHRC1 axis might broaden the application of the FAK-inhibitor VS-6063 in LUAD. Given the above, our results demonstrated that LINC00518 could work as a promising biomarker of prognosis and synergetic therapeutic target of FAK-inhibitor in LUAD.

## Materials and methods

### Patients and tissue samples

We obtained twenty primary lung adenocarcinoma tissues and adjacent normal tissues (ANTs) from the First Affiliated Hospital of Soochow University (Supplementary Table S3). There are no patients who received radiotherapy or chemotherapy before surgery. Two pathologists diagnosed the histological characteristics of the tissue, independently. And the study was approved by the Human Research Ethics Committee of the First Affiliated Hospital of Soochow University. Moreover, we obtained informed consent from all patients. Lung adenocarcinoma samples from The Cancer Genome Atlas (TCGA) (https://portal.gdc.cancer.gov/. Accessed 2 May 2020.) were also included in this study.

### Dataset Analysis

We screened the differentially expressed lncRNAs from the TCGA database with R version 3.63 (fold change=1.0, *P* < 0.05). The survival status and survival time of the patients were downloaded from the TCGA database (https://portal.gdc.cancer.gov/.) to obtain a prognostic model. Next, we combined the information about 309 patients with complete clinical data on TCGA-LUAD with the risk score, and the independent prognostic factor was analyzed by using the “survival” package of R software.

### The ceRNA network

We used the Cytoscape (v3.6.0) to construct the network of the LINC00518-miRNA-target gene to visualize their interactions. And we predicted the LINC00518/miRNA interaction by using miRcode (https://www.mircode.org/), and identified the target genes of the miRNAs by using miRDB (https://www.mirdb.org/), TargetScan (https://www.targetscan.org/), and miRTarBase (https://mirtarbase.mbc.nctu.edu.tw/).

### Materials

We purchased the cells from the Cell Bank of the Chinese Academy of Sciences in Shanghai, China, including A549, H1299 (lung adenocarcinoma), H460 (large cell carcinoma), H226 (lung squamous cell carcinoma), and BEAS-2B (human immortalized normal epithelial cells) cells. And cultured them in RPM1 1640 medium with 10% fetal bovine serum (Gibco, Carlsbad, CA, USA) at 37 °C in a 5% CO_2_ atmosphere.

### RNA interference

MiR-335-3p mimics, miR-335-3p inhibitors, and the related negative control (NC) were purchased from GenePharma (Suzhou, China). The GenePharma (Suzhou, China) directly synthesized the small interfering RNA (siRNA) and sequences corresponding to the target sequences for us. The siRNA constructs were listed in Supplementary Table S1. Next, insert short hairpin RNA (shRNA) and its corresponding control sequences into the lentivirus vector (GenePharma, Suzhou, China). To obtain cells stably expressing miR-335-3p or LINC00518 shRNA, lung adenocarcinoma cells were infected with lentiviruses. Then, we performed with Lipofectamine 2000 to transfect siRNA into cells according to the instructions of the manufacturer.

### Quantitative real-time PCR analysis

As we described previously, the detailed processes were performed [37]. And we listed the primers used in the study in Supplementary Table S2. We normalized the CT values of the gene mRNA levels to those of β-actin, the internal control. The ^△△^Ct method was applied to calculate the relative quantities of these mRNAs.

### RNA Binding Protein Immunoprecipitation (RIP)

According to the manufacturer’s protocol, the RIP kit (BersinBio, Guangzhou, China) was used to accomplish RIP analysis. Firstly, lyse A549 and H1299 cells were lysed by RIP lysis buffer. Secondly, we incubated the lysate products, pre-conjugated with anti-IgG or anti-Ago2 antibody, with magnetic beads at 4 °C for 12–16 h or overnight. Then, to obtain purified RNA, we used protease K to eliminate proteins. Last, the expression level of LINC00518 and miR-335-3p were determined by qRT–PCR. We performed each experiment in triplicate.

### Cell proliferation analysis

LUAD cells were cultivated into 96-well plates (3000 cells per well) treated with siRNA or plasmid and the cell counting was tested by Cell Counting Kit-8 (Dojindo, Shanghai, China). Cells were stained with Giemsa and counted to dectet cell proliferation by clonogenic assay.

### Transwell assays and wound healing assay

According to the manufacturer’s protocol in our previous work [20], we photographed and counted the cells in transwell plates and observe and measure the scratch by microscope.

### Cell cycle analysis

The cells were stained with a PI/RNase mixture from Beyotime Biotechnology (Shanghai, China), particularly in the dark, at 37 °C for 30 min to the cell cycle analysis. Then, the stained cells were tested in a FACSCalibur system (Beckman Coulter, Brea, CA, USA).

### Western blot analysis

The procedure followed our research before [20], and the antibodies in the study was as follows: anti-CTHRC1 (ab85739), anti-CyclinD1 (ab226977), anti-Integrin-αV (ab179475) (Abcam, London, UK), anti-MMP-9 (13677), anti-Fak (13009), anti-p-Fak (Ser397) (8556) (Cell Signaling Technology), and anti-Integrin β3 (A19073) (Abclonal, Wuhan, China). Anti-β-actin (CW0096M), anti-rabbit (CW0103), and anti-mouse (CW0102) secondary antibodies.

### Luciferase reporter assay

We inserted the fragments of the CTHRC1 3’-UTR and LINC00518 containing the binding site of miR-335-3p into the pMIR-REPORT vector. Then, we cotransfected with luciferase reporter plasmids and related oligonucleotides in A549 and H1299 cells. Plasmids with mutation-binding sites were used as controls. To detect the luciferase activity of reporter plasmids, we used the Dual-Luciferase Reporter Assay System purchased from the Promega.

### Co-immunoprecipitation (co-IP) assay

Lysed the cell for 30 min with 1 ml of RIPA buffer (Cell Signaling Technology, Danvers, MA, USA). Next, scraping to collect the cells and the protein in the lysates was incubated for more than 12 h with anti-CTHRC1 (Abcam, London, UK) or normal rabbit IgG antibody at 4 °C with rotation. Overnight, incubate the mixture with protein A/G beads or anti-c-Myc magnetic beads at 4 °C for 4 h. Boil the beads in the 2×SDS protein loading buffer, after washing with lysis buffer triples, and then subject them to Western blot analysis.

### Animal experiments

The female BALB/c nude mice (about 3–4 weeks old) was purchased from the Experimental Animal Center of Soochow University and bred under pathogen-free conditions. All animal experiments was in accordance with the Guide for the Care and Use of Experimental Animals Center of Soochow University. Aim to find the xenograft model of lung carcinoma, we subcutaneously inoculated a total of 3.0×10^6^ A549 cells into the flanks, and the female mice were randomly divided into three groups (five mice per group): LV-NC, sh-LINC00518–1, sh-LINC00518–2. Finally, determine the tumor volume (V) by using the Vernier caliper to measure the width (W) and length (L) and apply the following formula: V = (L × W^2^) × 0.5.

### Immunohistochemistry (IHC)

Tumors were collected to fixed and embedded to be made into paraffin sections. Then sections were incubate with primary antibodies against CTHRC1 (Abcam, London, UK) and integrin β3 (Abclonal, Wuhan, China) at 4 °C overnight which were followed by the incubation of the corresponding secondary antibodies and DAB chromogenic reaction and HE staining [38].

Membranous PSMA quantification was determined by modified H-Score (H-SCORE = ∑ (pi × i) = (percentage of weak intensity area ×1)+(percentage of moderate-intensity area ×2)+(percentage of strong intensity area ×3), to determine the overall percentage of mPSMA positivity across the entire stained tumor sample, yielding a range from 0 to 300 [39].

### Statistical analysis

All data are expressed as mean standard deviation values for at least three independent experiments. One-way analysis of variance (ANOVA) was followed by post hoc test using GraphPad Prism 8 software (GraphPad, San Diego, CA, USA) to determine the significant difference between two groups. Values of *(*p* <0.05) were considered statistically significant.

## Supplementary information

Supplemental

editing certificate

## Data Availability

The datasets used and analyzed in this study are available from the corresponding author on reasonable request.
