# Supplementary material for: Long noncoding RNA LINC00518 contributes to proliferation and metastasis in lung adenocarcinoma via the miR-335-3p/CTHRC1 Axis
Source: Cell Death Discov. 2022 Mar 4;8:98. doi: 10.1038/s41420-022-00905-w (PMC8897435; doi:10.1038/s41420-022-00905-w)

Supplementary 1: Table S1 The siRNA constructs were as follows

Name	(5′->3′)
si–NC	UUCUCCGAACGUGUCACGUTT
si–LINC00518–1	CCGGGCTAGATGGAACCTTAGTGAACTCGAGTTCACTAAGGTTCCATCTAGCTTTTT
si–LINC00518–2	CCGGCACCTCCAAAGTGACGACTTACTCGAGTAAGTCGTCACTTTGGAGGTGTTTTT
si–CTHRC1–1	CACAUUCAAUGGAGCUGAATT
si–CTHRC1–2	CGGAGUGUACAUUUACAAATT

Supplementary 2: Table S2 The sequences of primers used in this study

Name	Sequence (5′->3′)
LINC00518 forward	GTGAAAATCTGGCTACTCGTCCC
LINC00518 reverse	CTGACTTTTGCCACAGACTCCTG
CTHRC1 forward	GCATGCTGTCAGCGTTGGTA
CTHRC1 reverse	TCAATGGGAAGAGGTCCTGAA
β-actin forward	CACAGAGCCTCGCCTTTGC
β-actin reverse	ACCCATGCCCACCATCACG

The primer of miR-335-3p and U6 were synthesized by Ribobio (Guangzhou,China).

Supplementary 3: Table S3 The correlation between clinicopathological features and LINC00518 expression in 20 LUAD Patients.

Features	All cases	LINC00518 expression

	Total	Low	High	P value
**Total number**	20	5	15	0.035*
**Age**				
>60	16	4	12	0.416
≤60	4	1	3	
**Gender**				
male	16	4	12	0.416
female	4	1	3	
**Lymph node metastasis**				
with	8	2	6	0.173
without	12	3	8	
**Tumor size (cm)**				
>5	4	2	2	0.138
≤5	16	3	13	
**TNM stage**				
I+II	16	5	11	0.416
III+IV	4	0	4	

*P < 0.05

Supplementary 4: Table S4 The minimum lambda of the LASSO regression including 39 lncRNAs

ABCA9-AS1	AC015849.5	AC018529.1	AC020637.1	AC026462.3	AC027228.2

AC034223.1	AC048383.1	AC093904.3	AC106799.3	AC108136.1	AC139722.1
AL031599.1	AL035252.2	AL161431.1	AL161938.1	AL353746.1	AL731533.2
AP000438.1	CHODL-AS1	CU639417.4	FAM83A-AS1	LINC00211	LINC00518
LINC00867	LINC01116	LINC01221	LINC01776	LINC01811	LINC02310
LINC02576	MED4-AS1	NKAIN3-IT1	NPSR1-AS1	PTGES2-AS1	SAMSN1-AS1
SATB2-AS1	TFAP2A-AS1	ZBTB20-AS3			

Supplementary 5: Table S5 The result of IHC and clinicopathological features in 8 LUAD Patients.

	**Gender**	**Age**	**TNM stage**	**Tiusse**	**Positive Area percent (%)**						**H-score**	
					CTHRC1			Integrin β3				
					1 week	2 week	3 week	1 week	2 week	3 week	CTHRC1	Integrin β3
				Tumor	76.83769687	8.386218237	0.141311888	68.76631	3.205367	0.111815	14.63477	75.51249
1	Male	74	T1N0M0	Normal	47.64284305	9.356302189	0.302452495	19.25795	0.530035	0	42.6984	20.31802
				Tumor	71.86653856	12.16409002	0.005822925	29.54856	0	0	15.96355	29.54856
2	Male	58	T1N0M0	Normal	59.35152405	6.368189276	0.112252828	6.189555	0	0	34.16803	6.189555
				Tumor	74.72647206	7.232033414	0.313251918	63.64573	1.441813	0	17.72824	66.52935
3	Female	65	T2N0M0	Normal	80.7640112	6.846071666	0.098172263	62.40538	0.756939	0	12.29174	63.91926
				Tumor	92.66470732	5.963752143	0.036737693	81.38298	0.531915	0	1.334803	82.44681
4	Female	67	T1N0M0	Normal	66.45116601	14.43084738	0.230893558	33.33333	2.259887	0	18.88709	37.85311
				Tumor	58.33751883	5.713209442	0.037669513	34.65553	1.670146	0	35.9116	37.99582
5	Male	50	T2N0M0	Normal	60.0251076	18.27474892	1.380918221	37.83784	0.675676	0	20.31923	39.18919

				Tumor	66.14785992	5.7230869	0.859273671	52.63158	2.631579	0.877193	27.26978	60.52632
6	Male	72	T2N0M0	Normal	61.13851419	11.95892893	0.342258909	40.56902	0.948367	0	26.5603	42.46575
				Tumor	48.89519259	5.98686175	0.01492983	31.5678	0.423729	0	45.10302	32.41525
7	Female	65	T1N0M0	Normal	76.62427462	8.495857728	0.173187029	52.27092	1.533865	0.01992	14.70668	55.39841
				Tumor	51.52894652	7.872350209	0.018393342	27.53404	2.420575	0.151286	40.58031	32.82905
8	Male	64	T2N1M0	Normal	66.90396804	15.42534018	1.057342825	30.77314	1.220753	0.178026	16.61335	33.74873

Supplementary 6:

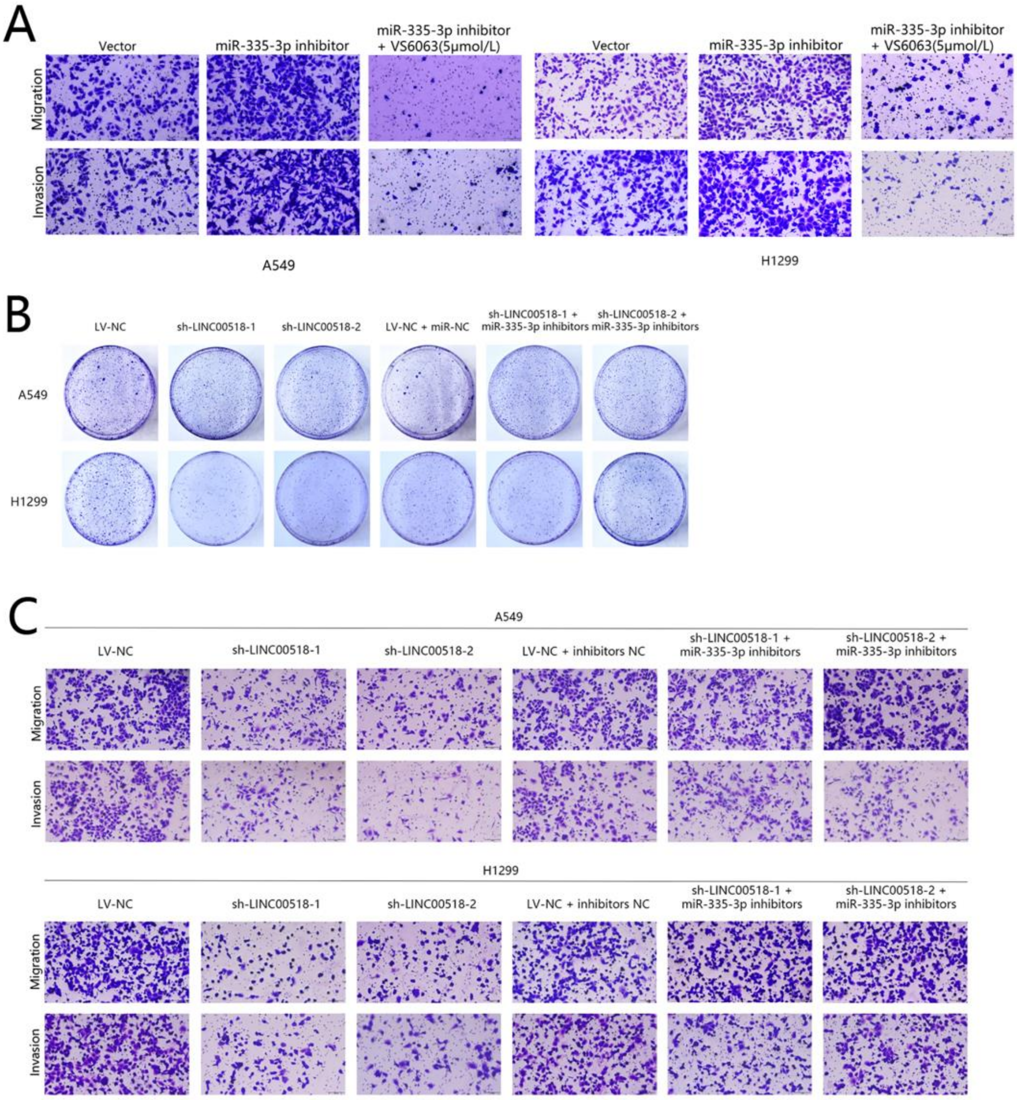

Figure S1. (A) As is shown in Transwell assays that miR-335-3p inhibited cells migration and invasion following VS6063 (5 μmol/L). (B) Colony formation analysis was aimed to detect the proliferation of A549/H1299 cells transfected with LINC00518 shRNA, LINC00518 shRNA plus miR-335-3p inhibitor, or the control. (C) As is shown in the transwell assay the effect on LUAD cell migration and invasion following transfection is described before.

Supplementary 7:

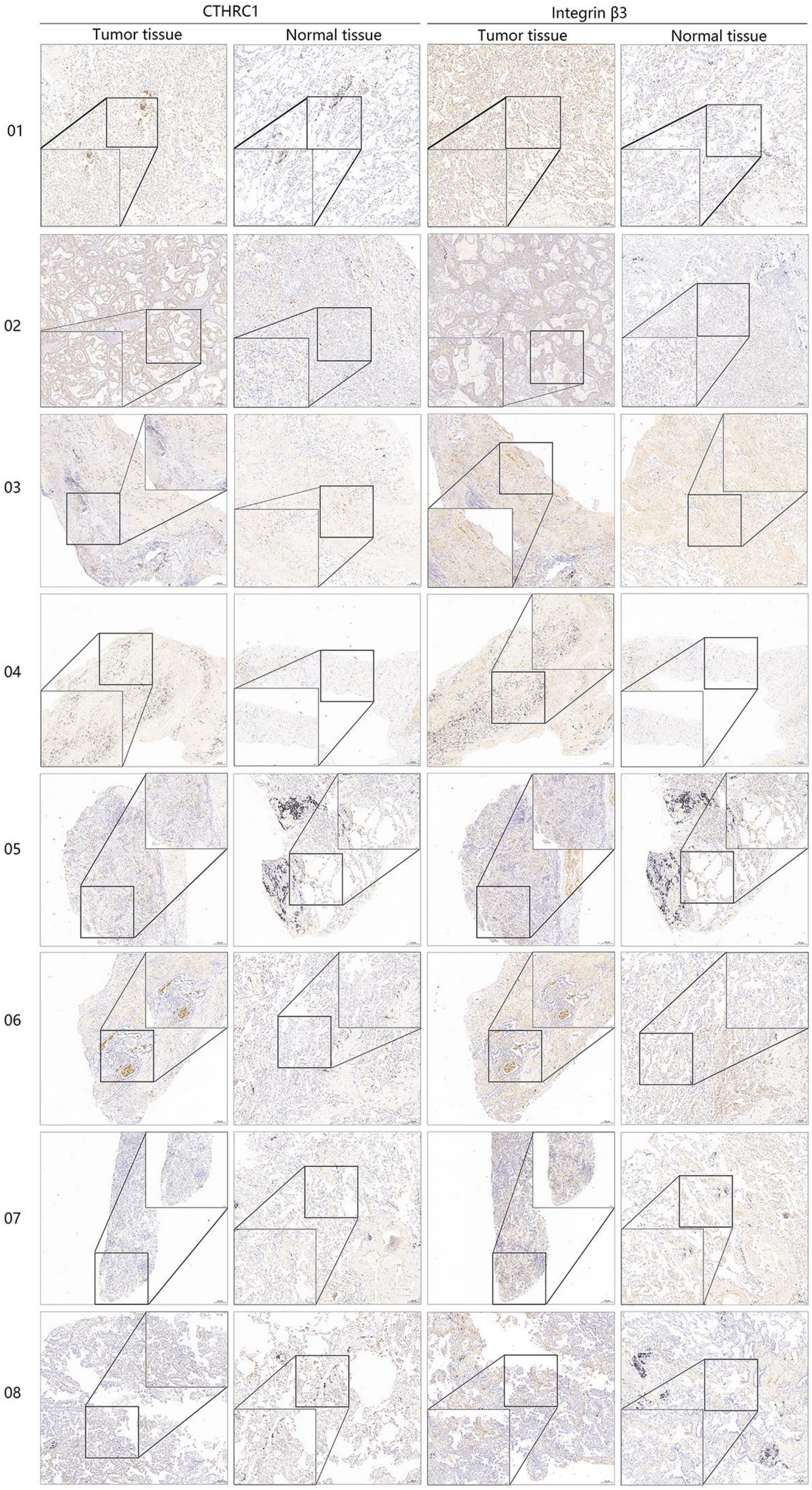

Figure S2. The NSCLC samples were immunostained with anti-CTHRC1 and anti-integrin β3 antibodies (scale bar, 100 μm, 50 μm).

Supplementary 8: The original western bloting

Fig.5C

CTHRC1

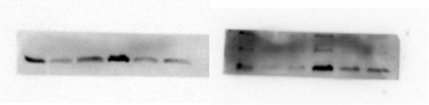

β3

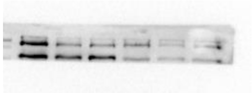

Pfak

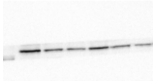

MMP9

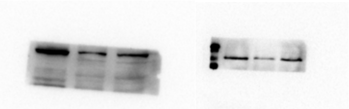

FAK

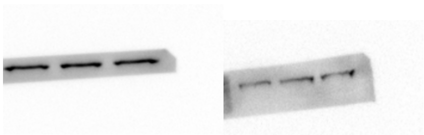

CD1

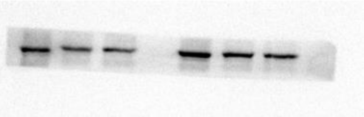

Actin

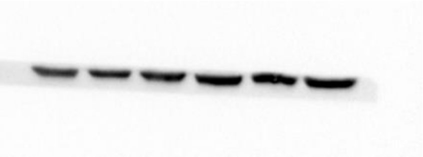

Fig.5D

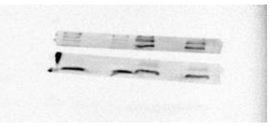

Fig.5J

Actin

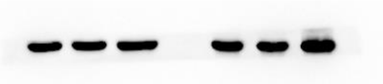

CTHRC1

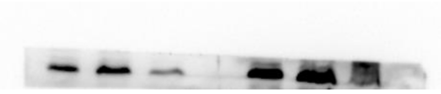

Pfak

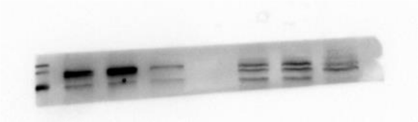

Fak

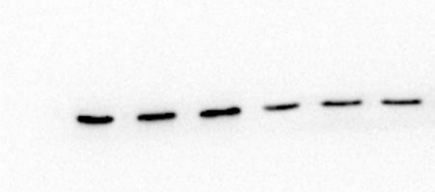

Fig.6E

actin

CTHRC1

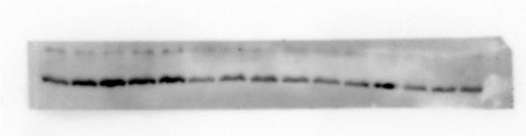

Fig.7E

CTHRC1

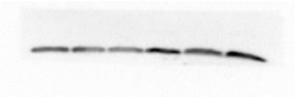

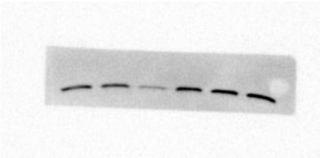

ACTIN

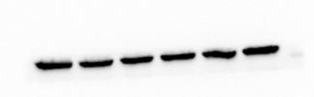

Supplement: Supplementary file 1 — Supplemental [file 41420_2022_905_MOESM1_ESM.pdf]
