# Supplementary material for: Long noncoding RNA LINC00518 contributes to proliferation and metastasis in lung adenocarcinoma via the miR-335-3p/CTHRC1 Axis
Source: Cell Death Discov. 2022 Mar 4;8:98. doi: 10.1038/s41420-022-00905-w (PMC8897435; doi:10.1038/s41420-022-00905-w)
Supplement: Supplementary file 2 — editing certificate [file 41420_2022_905_MOESM2_ESM.pdf]

SPRINGER NATURE

Author Services

Editing Certificate

This document certifies that the manuscript

Long noncoding RNA LINC00518 Contributes to Proliferation and Metastasis in Lung adenocarcinoma via the miR-335-3p/CTHRC1 Axis

prepared by the authors

Ruoyi Shen, Xin Cai, Dan Shen, Ruochen Zhang, Weijie Zhang, Yang Zhang, Yue Li, Anqi Wang, Yuanyuan Zeng, Jianjie Zhu, Zeyi Liu, Jian-an Huang

was edited for proper English language, grammar, punctuation, spelling, and overall style by one or more of the highly qualified native English speaking editors at SNAS.

This certificate was issued on **August 30, 2021** and may be verified on the SNAS website using the verification code **0708-E818-C182-F982-10DE** .

Neither the research content nor the authors' intentions were altered in any way during the editing process. Documents receiving this certification should be English-ready for publication; however, the author has the ability to accept or reject our suggestions and changes. To verify the final SNAS edited version, please visit our verification page at secure.authorservices.springernature.com/certificate/verify.
If you have any questions or concerns about this edited document, please contact SNAS at support@as.springernature.com.

SNAS provides a range of editing, translation, and manuscript services for researchers and publishers around the world.
For more information about our company, services, and partner discounts, please visit authorservices.springernature.com.
